# Das Mikrobiom bei Erkrankungen der Nase und der Nasennebenhöhlen

**DOI:** 10.1007/s00106-025-01661-w

**Published:** 2025-08-15

**Authors:** Tina Bartosik

**Affiliations:** Universitätsklinik für Hals‑, Nasen- und Ohrenkrankheiten, Medizinische Universität Wien, Währinger Straße 18–20, 1090 Wien, Österreich

**Keywords:** Nasales Mikrobiom, Chronische Rhinosinusitis, Geringe Biomasse, N‑ERD, Probengewinnung, Nasal microbiome, Chronic rhinosinusitis, Low biomass, N‑ERD, Sample collection

## Abstract

Das nasale Mikrobiom wird als Synonym für die Summe und die Diversität der Bakterien im Nasenbereich verwendet. Während das Darmmikrobiom in den letzten Jahren aufgrund von Fortschritten bei den Diagnosetechnologien im Mittelpunkt der Mikrobiomforschung stand, richtet sich die Aufmerksamkeit nun zunehmend auf mikrobielle Ökosysteme in anderen Körperregionen einschließlich der Atemwege. Prinzipiell entspricht eine vermehrte und stabile Vielfalt (α-Diversität) einer „gesunden“ Flora, und *vice versa* zeigt sich eine verminderte bakterielle Diversität z. B. im Krankheitsbild einer chronischen Rhinosinusitis. Ob dies als Ursache für die Entstehung der Krankheit oder als deren Konsequenz zu werten ist, kann anlässlich der aktuellen Datenlage noch nicht sicher beantwortet werden. Bedingt durch die geringe mikrobielle Biomasse in der Nase und die daraus resultierenden erschwerten Analysemöglichkeiten, aber auch aufgrund der uneinheitlichen Probengewinnung und Datenauswertung wegen fehlender Guidelines ist eine Interpretation der aktuellen heterogenen Studienlage erschwert. Dies unterstreicht die Notwendigkeit konsistenter, leitliniengestürzter Forschungsprotokolle in diesem neuen Bereich.

## Abstract (en)

The nasal microbiome refers to the composition and diversity of bacterial communities residing in the nasal cavity. While the gut microbiome has been the primary focus of microbiome research in recent years, driven by advances in diagnostic technologies, increasing attention is now being directed toward microbial ecosystems in other regions of the body, including the respiratory tract. In general, a high and stable microbial diversity (α-diversity) is considered indicative of a “healthy” microbial community. Conversely, reduced bacterial diversity has been associated with clinical conditions, such as chronic rhinosinusitis (CRS); however, it remains unclear whether such microbial alterations are a cause or consequence of the disease, as current evidence does not yet allow for definitive conclusions. Interpreting existing studies on the nasal microbiome is challenging due to several factors: the low microbial biomass in the nasal cavity, the technical difficulty of accurately analyzing such samples, and the lack of standardized methodologies for sampling and data interpretation. These limitations contribute to the heterogeneity of current findings and highlight the need for consistent, guideline-based research protocols in this emerging field.

## Das Mikrobiom der Nase

Das nasale Mikrobiom umfasst die Gesamtheit aller Mikroorganismen (Bakterien, Archaeen, Viren, Pilze etc.) im Naseneingang, in der Nase und in den Nasennebenhöhlen. Der Begriff wird fälschlicherweise oft lediglich für die Gesamtheit aller Bakterien verwendet, auf welcher das Hauptaugenmerk der aktuellen Forschung liegt.

Im Zuge des Human Microbiome Project (HMP) wurde im Laufe von 10 Jahren (2007–2016) das Mikrobiom des Menschen, systemisch geordnet nach Körperregionen, an gesunden Probanden untersucht und dokumentiert [1]. Im Zuge der zweiten Phase dieser Langzeitstudie wurden dann die funktionelle Rolle und die Differenzen zwischen Gesundheit und Krankheit analysiert. Diese Arbeit gilt als Meilenstein in der Mikrobiomforschung und dient als Referenzdatenbank für das Mikrobiom bei gesunden Erwachsenen [2].

Das nasale Mikrobiom besteht typischerweise aus verschiedenen Bakteriengattungen, die in ihrer Zusammensetzung individuell variieren. Zu den am häufigsten nachgewiesenen Gattungen zählen Corynebacterium, Staphylococcus (darunter *S. aureus* und *S. epidermidis*), Propionibacterium (insbesondere *Cutibacterium acnes*), Dolosigranulum und Moraxella. Das nasale Mikrobiom weist jedoch eine viel niedrigere Diversität auf als z. B. der Darm, die Haut oder die Mundhöhle [3].

Da die Nase aber im ständigen Kontakt mit der Umwelt (Luft, Staub, Allergene etc.) steht und als Eintrittspforte für Pathogene gilt, wird ihr eine Rolle in der lokalen Immunabwehr zugesprochen. Es besteht die Hypothese, dass die mikrobielle Gemeinschaft das Wachstum von pathogenen Keimen durch die schützende Rolle von Corynebacterium und Dolosigranulum hemmt. Zudem wird ihr eine modulierende Wirkung auf immunologische Prozesse zugesprochen, indem sie die Balance zwischen immunologischer Toleranz und aktiver entzündlicher Abwehrreaktion unterstützt. Zusätzlich soll sie die Barrierefunktion der mukosalen Oberfläche, ähnlich wie die Funktion der Darmmikrobiota, stärken [4].

## Probengewinnung

Die Probenentnahme zur Analyse des nasalen Mikrobioms kann auf verschiedene Weise erfolgen. Am häufigsten angewandt werden Nasenabstriche („swabs“). Sie sind minimal-invasiv und eignen sich gut für die Analyse bakterieller DNA und RNA. Dabei wird mit einem Wattestäbchen Material entweder aus dem Naseneingang oder aus tiefer gelegenen Bereichen wie dem mittleren Nasengang entnommen.

Studien mit Proben aus verschiedenen Regionen desselben Patienten zeigen zwar Unterschiede im Mikrobiom zwischen den einzelnen Nebenhöhlen, jedoch ist die Variabilität zwischen verschiedenen Personen deutlich größer als die innerhalb einer Person. Rezente Studienergebnisse zeigen, dass Proben aus dem Antrum nasi und dem mittleren Nasengang das Mikrobiom der Nebenhöhlen widerspiegeln, welches in der Regel nur intraoperativ untersucht werden kann. Daher kann auf eine invasive Probengewinnung verzichtet werden [5].

Eine alternative Methode ist die Nasenspülung (Lavage), bei der die Nase mit isotoner Kochsalzlösung gespült und die zurückfließende Flüssigkeit gesammelt wird. Dabei kann es jedoch zu Schwierigkeiten bei der Mitarbeit der Probanden kommen, da es ihnen möglicherweise schwerfällt, die Lösung in der Nase zu behalten, ohne sie versehentlich zu schlucken.

Eine weitere Möglichkeit besteht in der Biopsie der Nasenschleimhaut, welche jedoch mit einer größeren Invasivität einhergeht. In einer kleinen Studie konnte gezeigt werden, dass Schleimhautbiopsien und Schleimhautabstriche bei Patienten mit chronischer Rhinosinusitis (CRS) eine vergleichbare bakterielle Diversität und Zusammensetzung aufweisen. Auch hier war die Variation zwischen verschiedenen Personen größer als die Unterschiede zwischen den beiden Probenarten bei ein und derselben Person [6].

## Analysen und Beurteilung

Das nasale Mikrobiom weist im Vergleich zum Darm oder zur Haut eine viel niedrigere Biomasse auf, wodurch die Gefahr der Verfälschung durch Kontaminationen stark erhöht ist. Zusätzlich besteht eine hohe Wirts-DNA-Kontamination. Für die Forschung im Bereich des nasalen Mikrobioms ist daher eine kontaminationsarme Probenentnahme durch ein bevorzugt kleines und konstantes Team in denselben Räumlichkeiten empfohlen. Außerdem sollten Kontrollproben aus der Umgebungsluft entnommen werden, um diese Verunreinigungen aus den Analysedaten zu subtrahieren.

Der derzeitige Goldstandard im Bereich der bakteriellen Diversitätsanalyse ist die Amplikonsequenzierung des 16S-Ribosomen-RNA-Gens (16S-rRNA). Zur Sequenzierung der gesamten DNA und somit Auswertung der Genfunktion steht die teurere und aufwendigere metagenome Sequenzierung zur Verfügung. Diese Methode liefert jedoch auch Daten zu Viren, Pilzen, Archaeen etc. sowie zur mitochondrialen DNA des Wirts, wodurch ein höherer Aufwand in Form von Kosten und Datenaufarbeitung zur Aufschlüsselung der Informationen notwendig ist [7].

Sogar renommierte Journale haben Publikationen veröffentlicht, welche keine Angaben zu Kontrollen im Zuge der Probengewinnung, DNA-Extraktion oder 16S-rRNA-Gen-Amplikon-Präparation vorgebracht haben. Aus kontaminierten Proben und somit erweiterten α‑Diversitäten wurden daher fälschlicherweise Schlussfolgerungen bezüglich der Rolle des nasalen Mikrobioms in Bezug auf die Diagnosestellung der CRS gezogen [8].

Für die Forschung in diesem Gebiet bedeutet dies daher, dass eine kontaminationsarme Probenentnahme, eine standardisierte Laborhygiene sowie bioinformatische Kontrollen und Aufarbeitung essenziell sind. All diese Faktoren sollten bei der Interpretation von Mikrobiomstudien durch die Leser berücksichtigt werden [9].

## Mikrobiom bei chronischer Rhinosinusitis

Die CRS ist eine chronisch-entzündliche Erkrankung der Nase und der Nasennebenhöhlen, die anhand des Vorliegens von Nasenpolypen in 2 Phänotypen, nämlich CRSsNP (ohne Polypen) und CRSwNP (mit Polypen), eingeteilt werden kann. Eine Sonderform stellt die N‑ERD („NSAID-exacerbated respiratory disease“) dar, bei der zusätzlich Asthma und eine Überempfindlichkeit gegenüber nichtsteroidalen Antirheumatika (NSAR) vorliegen.

Die bisher veröffentlichen Übersichtsarbeiten fassten eine veränderte mikrobielle Zusammensetzung (Dysbiose) der Nasen- und Nasennebenhöhlenflora mit einhergehender verminderter mikrobieller Diversität und Zunahme pathogener Bakterien (v. a. *Staphylococcus aureus*) zusammen [10]. Bei näherer Betrachtung fällt jedoch auf, dass die Studienergebnisse sehr heterogen sind. Diese Varianz kann zum einen den verschiedenen Subtypen der CRS mit jeweils unterschiedlichen pathophysiologischen Mechanismen zugeordnet werden. Andererseits sind auch die unterschiedlichen Studiendesigns mit Variablen der Probengewinnung und der weiteren Aufarbeitung hierfür ursächlich.

Zusammengefasst zeigen sich bei Patienten mit CRSwNP und N‑ERD eine vermehrte Besiedelung mit Staphylokokken und ein Rückgang von den als protektiv gewerteten Corynebakterien. Es ist aber bis heute noch unklar, ob dies die Folge der Erkrankung ist oder eine ursächliche Rolle in der Entstehung der CRS einnimmt [11].

## Bedeutung der Therapien der CRS

Für die CRS stehen im Moment mehrere Therapieoptionen zur Verfügung. Neben der Lokaltherapie mit Kortikosteroiden oder Spülungen (z. B. Kochsalz oder Xylitol) werden zusätzlich Operationen (funktionelle endoskopische Sinuschirurgie) und mittlerweile auch Biologika (z. B. Dupilumab, Mepolizumab etc.) angewandt. Eine antibiotische Therapie (lokal oder systemisch) wird lediglich bei akuten Exazerbationen der CRS bzw. einer akuten bakteriellen Sinusitis empfohlen. Eine weitere Indikation für eine antibiotische Therapie besteht in einer therapieresistenten chronischen Nicht-Typ-2-Rhinosinusitis, welche durch eine fehlende Eosinophilie und IgE(Immunglobulin E)-Werte im Normbereich gekennzeichnet ist [12]. Die Auswirkungen dieser angeführten Therapien wurden bisher in eher kleinen Studien mit geringer Probandenzahl evaluiert und brachten gegensätzliche Erkenntnisse zutage.

In einer rezenten Studie mit 3 Behandlungsgruppen (topische und systemische Kortikosteroide, Doxycyclin) konnten klinische Erfolge der CRS in den Kortisongruppen, jedoch unabhängig von den Veränderungen des nasalen Mikrobioms, nachgewiesen werden. Ein signifikanter mikrobieller Effekt wurde lediglich bei der topischen Budesonidanwendung in Form eines vorübergehenden Anstiegs der bakteriellen Diversität nach Behandlungsbeginn gezeigt. Diese Reaktion hielt jedoch unabhängig von den positiven klinischen Parametern nur für 3 Wochen an [13].

In einer Metaanalyse konnte zudem festgestellt werden, dass kurzfristiger oder kumulativer Antibiotikagebrauch keine signifikanten Auswirkungen auf die bakterielle Zusammensetzung in den Nasennebenhöhlen hatte [14]. Auch Studien mit Doxycyclin oder Roxithromycin zeigten keine relevanten Veränderungen hinsichtlich Diversität oder Bakterienreichtum bei Patienten mit CRS [15, 16].

Der Einfluss der funktionellen endoskopischen Sinuschirurgie auf das Mikrobiom ist ebenfalls uneinheitlich – in manchen Studien wird von einer Zunahme, in anderen von einer Abnahme der bakteriellen Diversität berichtet. Auch hier wird dies auf unterschiedliche Studienprotokolle mit Differenzen im Zeitpunkt der Probenentnahme und der Nachbehandlung zurückgeführt [17, 18].

## Fazit für die Praxis

- Die aktuelle Studienlage zeigt kontroverse Befunde und erschwert die Interpretation der Ergebnisse. Aufgrund der niedrigen Biomasse und des daraus resultierenden Risikos der Kontaminationen ist die Vorgabe von internationalen Guidelines und Sicherheitsmaßnahmen notwendig.
- Für die Planung von Mikrobiomstudien sollte eine enge Zusammenarbeit eines konstanten Teams und erfahrener Bioinformatiker priorisiert werden. Es werden standardisierte Protokolle für Probenentnahme, Analyse und Interpretation empfohlen.
- Zurzeit lassen die Studienergebnisse noch keine Konsequenzen bezüglich der Therapieplanung der chronischen Nasenerkrankungen zu. Des Weiteren wird eine individuelle Mikrobiomanalyse noch nicht als Diagnosetool befürwortet.
- Es wird jedoch im Bereich der Probiotikatherapien geforscht, und personalisierte Therapien in Form von mikrobiombasierten Behandlungen werden als mögliche zukünftige medizinische Versorgungsoption evaluiert.
